# An Unseen Challenge: Mycotic Pseudoaneurysm Emerges in the Wake of Everolimus-Eluting Stent Deployment

**DOI:** 10.7759/cureus.54363

**Published:** 2024-02-17

**Authors:** Iyad Farouji, Ahmad Aboarqoub, Arwa Battah, Hussein Shaqra

**Affiliations:** 1 Cardiology, Saint Michael's Medical Center, Newark, USA; 2 Cardiology, St. Claire HealthCare, Morehead, USA; 3 Internal Medicine, Saint Michael's Medical Center, Newark, USA; 4 Cardiology, St. Mary's General Hospital, Newark, USA

**Keywords:** drug-eluting stent, coronary artery pseudoaneurysm, pci complication, pseudoaneurysm clipping, mycotic pseudoaneurysm

## Abstract

This case report presents a unique instance of mycotic pseudoaneurysm in the proximal right coronary artery (RCA) following percutaneous coronary intervention (PCI) in a 75-year-old male with a complex medical history. Despite successful initial intervention and resolution of bacteremia, the patient presented three months later with recurrent anginal symptoms. The diagnostic evaluation revealed a mycotic pseudoaneurysm in the RCA, leading to surgical clipping and graft implantation. The successful surgical outcome underscores the critical role of early recognition and intervention in enhancing patient survival. This case contributes valuable insights into the diagnostic intricacies and therapeutic nuances of mycotic pseudoaneurysm, reinforcing the importance of maintaining a heightened index of suspicion, particularly in patients with a history of coronary interventions.

## Introduction

Mycotic pseudoaneurysm of coronary arteries following percutaneous coronary intervention (PCI) is an exceptionally rare complication, presenting a diagnostic challenge due to its infrequent occurrence and the potential absence of bacteremia [1]. Early identification is crucial, as these cases often demand swift surgical interventions to mitigate the associated high mortality [2]. In this report, we present a unique case of a mycotic pseudoaneurysm in the proximal right coronary artery (RCA) that developed after an everolimus-eluting stent placement during PCI. Despite the rarity of this complication, its implications are profound, necessitating prompt recognition and intervention.

## Case presentation

A 75-year-old male, with a notable medical history including hypertension, dyslipidemia, paroxysmal atrial fibrillation, and peripheral vascular disease treated with percutaneous transluminal angioplasty (PTA) of the left common iliac and common femoral artery, presented to the Emergency Department due to chest pain. He smokes tobacco and has no history of intravenous drug abuse (IVDA). He described the chest pain as sudden-onset, characterized by heaviness and retrosternal discomfort radiating to the left arm. The pain, initially exacerbated by exertion, had progressed to occurring at rest. Over the preceding two weeks, the patient reported accompanying symptoms of nausea, diaphoresis, subjective fever, and chills, with no other pertinent findings on the review of systems.

The physical examination revealed a temperature of 100°F. Blood work showed normal troponins, leukocytosis, elevated acute inflammatory markers, and two bottles containing methicillin-sensitive *Staphylococcus aureus* (MSSA) were detected in the blood cultures (refer to Table 1). Comprehensive investigations were conducted following the positive blood cultures, encompassing a thorough physical examination, chest X-ray, and abdominal computerized tomography, all yielding negative results. He has been started on cefazolin. A transthoracic echocardiogram (TTE) found no evidence of vegetation, while a pharmacological nuclear stress test indicated reversible ischemia in the inferior wall.

**Table 1:** Laboratory results. MSSA: methicillin-resistant *Staphylococcus aureus*

Test	Result	Reference
White blood cells (WBC)	13,000 cells/mm^3^	4,400-11,000 cells/mm^3^
C-reactive protein (CRP)	52.4 mg/L	0.0–8.0 mg/L
Erythrocyte sedimentation rate (ESR)	42 mm/hr	<20 mm/hr
Troponins-I	0.01 ng/mL	<0.04 ng/mL
Blood culture	Positive MSSA - 2/2 bottles	Negative

Subsequently, the patient underwent PCI for multivessel coronary heart disease, targeting the ostial and proximal RCA. Simultaneously, appropriate antibiotic therapy was administered, resulting in the clearance of bacteremia and gradual symptom resolution, leading to discharge home.

**Figure 1:**
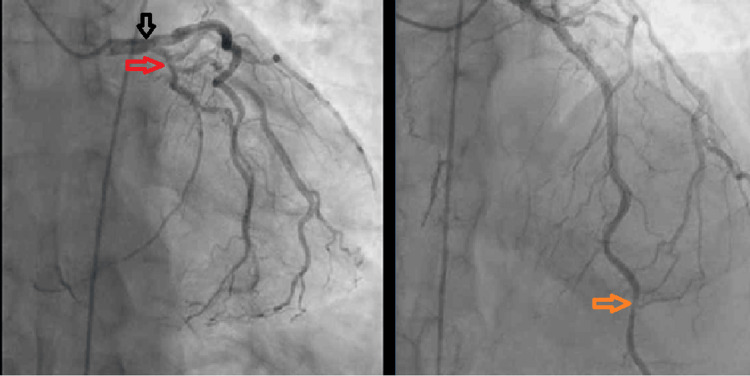
Coronary angiogram showed moderate distal left main disease (black arrow), severe proximal Lcx (red arrow), and distal LAD disease (orange arrow) with a patent mid LAD stent. Lcx: left circumflex, LAD: left anterior descending

**Figure 2:**
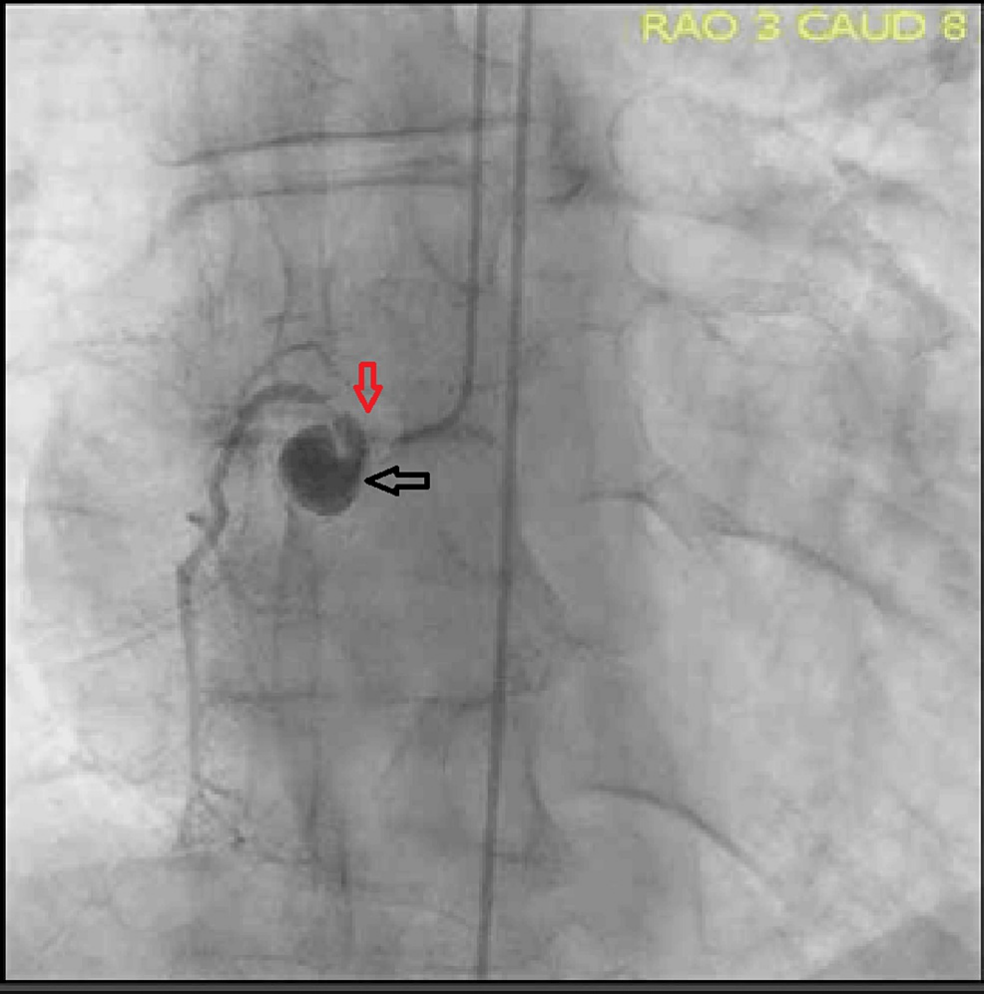
Coronary angiogram showing pseudoaneurysm of ostial RCA (black arrow) with subtotal occlusion of the proximal portion (red arrow). RCA: right coronary artery

Three months later, the patient returned to the Emergency Department with recurrent anginal symptoms despite receiving appropriate medical therapy including aspirin 81 mg, clopidogrel 75 mg, metoprolol succinate 50 mg, and lisinopril 20 mg. Blood cultures were repeated and were negative. Coronary angiography revealed moderate distal left main disease, severe proximal left circumflex artery disease, severe distal left anterior descending (LAD) artery disease, and a pseudoaneurysm/aneurysm involving the ostial portion of the RCA with severe distal obstruction (refer to Figures 1-2).

The definitive diagnosis of pseudoaneurysm versus aneurysm was not established until the patient underwent surgery. During the surgical intervention, the diagnosis of mycotic pseudoaneurysm was confirmed by both the gross shape and later histopathological examination and tissue culture. Subsequently, the patient underwent clipping of the pseudoaneurysm without resection. In addition, implantation of three grafts (left internal mammary artery (LIMA) to LAD, saphenous vein graft (SVG) to obtuse marginal (OM1/OM2), and SVG to RCA) was performed. Following the surgical intervention, the patient was discharged home in good condition, experiencing total resolution of the ischemic symptoms.

## Discussion

An infrequent occurrence, coronary artery aneurysm (CAA) is characterized by the expansion of the coronary artery, surpassing 50% of the reference vessel diameter [3]. If the diameter of CAAs exceeds four times the reference vessel diameter or is greater than 8 mm, they are classified as giant [4]. Epicardial coronary arteries host CAAs predominantly in the proximal and middle sections of the RCA (68%). The proximal LAD exhibits CAAs in 60% of cases and the left circumflex (LCx) in 50%. Notably, CAAs in the left main stem (LMS) are exceptionally rare [5].

The rising prevalence of drug-eluting stents (DESs) has led to an escalating number of reports indicating stent-induced coronary aneurysms emerging months or even years post-intervention. The occurrence of coronary aneurysms after intervention is relatively rare, with a documented incidence ranging from 0.3% to 6.0%. It is noteworthy that a majority of these aneurysms are, in fact, pseudoaneurysms rather than authentic aneurysms [6]. Nevertheless, mycotic (infective) CAAs are exceptionally uncommon, representing less than 3% of the total cases of coronary aneurysms [1].

Typically linked to infective endocarditis (IE), mycotic aneurysms often manifest in patients with sepsis, particularly those who are immunocompromised. The predominant causative pathogens identified are *Staphylococcus aureus* and *Streptococcus viridans* [2]. The involvement of coronary stents in the development of mycotic coronary aneurysms is on the rise, and the associated organisms are akin to those observed in IE [2].

Aneurysms of infectious origin following intracoronary stent placement can be attributed to various mechanisms, including contamination during stent delivery, transient bacteremia originating from skin flora through access-site hematomas, pseudoaneurysms, delayed bleeding, extended arterial sheath insertion, and multiple procedures conducted from the same access site within a brief timeframe [7].

The incidence of mycotic aneurysms is more pronounced with the use of bare-metal stents, but this trend is diminishing as the prevalence of DESs increases [8]. Among the DESs, sirolimus (Cypher) exhibits the highest association with mycotic aneurysms [9]. Its mechanism of action involves mitigating the innate inflammatory response to bacterial invasion by suppressing IL-10 production. While the literature review reveals only a few instances of mycotic aneurysms associated with everolimus DESs, the most likely mechanism encountered mirrors that observed in the sirolimus era through the mammalian target of the rapamycin (mTOR) cascade. Another proposed mechanism is a hypersensitivity reaction to the metallic stent, the polymer, or the eluting drug [9].

The occurrence of mycotic aneurysms following PCI without concurrent IE is exceptionally rare, thereby posing a significant diagnostic challenge, particularly in patients with bacteremia of unknown origin, as observed in our case [10]. Timely identification is imperative due to the elevated mortality associated with this condition in the absence of surgical intervention, as antibiotic treatment proves ineffective under such circumstances.

Aneurysms smaller than 1-2 cm may potentially resolve with antibiotic therapy; nevertheless, larger ones have the propensity to expand and eventually rupture, leading to cardiac tamponade and sudden death. Consequently, larger aneurysms should be either excised or excluded from circulation, with simultaneous revascularization of the distal coronary artery [11]. Surgical interventions, such as excision of the mycotic coronary aneurysm along with distal bypass of the artery, are commonly performed. Alternatively, procedures like deroofing and debridement of the aneurysm, combined with arterial ligation and distal bypass, may be undertaken. While some authors advocate percutaneous placement of covered stents for treating mycotic CAAs, surgical intervention remains the gold standard of care unless the patient is considered at a higher risk for surgery [12].

The occurrence of mycotic aneurysms following PCI without concurrent IE is exceptionally rare. The literature contains limited reports on this condition, with the predominant cases involving the use of sirolimus-eluting stents [13], thereby posing a significant diagnostic challenge, especially in patients with bacteremia of unknown origin, as observed in our case. Early recognition is vital due to the elevated mortality associated with this condition in the absence of surgical intervention, as antibiotic treatment proves ineffective under such circumstances. Our patient presented with anginal symptoms over three months after proximal RCA stent implantation, coinciding with transient bacteremia within a week of the intervention. While the patient was infection-free at the time of mycotic aneurysm diagnosis, the transient bacteremia post-stent implantation may pose an increased risk of mycotic pseudoaneurysm formation.

## Conclusions

The case report highlights a rare occurrence of a mycotic pseudoaneurysm in the proximal RCA following PCI in a 75-year-old male with multiple comorbidities. Despite an initially successful intervention and the clearance of bacteremia, the patient experienced recurrent anginal symptoms three months later. A diagnostic evaluation revealed a mycotic pseudoaneurysm in the RCA, leading to surgical clipping and graft implantation. The rarity of mycotic pseudoaneurysms, the associated diagnostic challenges, and the crucial importance of early surgical interventions are emphasized. This case underscores the necessity for heightened vigilance, especially in patients with transient bacteremia post-PCI, and provides valuable insights into the management of this uncommon but potentially life-threatening complication.
